# Pediatrician's knowledge on the management of the infant who cries excessively in the first months of life

**DOI:** 10.1590/0103-0582201432218713

**Published:** 2014-06

**Authors:** Ana Carolina C. Marcon, Mário César Vieira, Mauro Batista de Morais

**Affiliations:** 1 Hospital Pequeno Príncipe, Curitiba, PR, Brasil; 2 Escola Paulista de Medicina da Universidade Federal de São Paulo (Unifesp), São Paulo, SP, Brasil

**Keywords:** crying, infant, health education

## Abstract

**OBJECTIVE:**

To evaluate the attitude, the practice and the knowledge of pediatricians regarding the management of the infant who cries excessively in the first months of life.

**METHODS:**

Descriptive cross-sectional study that enrolled pediatricians (n=132) randomly interviewed at a Pediatric meeting in Brazil, in August 2012. The data were collected by a self-administered standardized form after reading the hypothetical case of an infant who cried excessively.

**RESULTS:**

The majority of the participants were females, the mean age was 39 years and the average mean time working in the specialty was 14 years; 52.2% were Board Certified by the Brazilian Society of Pediatrics. The diagnosis most often considered was gastroesophageal reflux disease (62.9%), followed by infant colic (23.5%) and cow's milk allergy (6.8%). The diagnostic test most frequently mentioned was 24-hour esophageal pH-monitoring (21.9%). The medications most frequently indicated were domperidone (30.3%), the combination of domperidone with ranitidine (12.1%) and paracetamol (6%).

**CONCLUSIONS:**

In the approach of the infant who cries excessively, diagnostic tests are frequently requested and unnecessary medical treatment is usually recommended.

## Abstract (es)

**OBJETIVO:**

Evaluar la actitud, la práctica y el conocimiento de pediatras sobre el manejo del lactante que llora excesivamente durante los primeros meses de vida.

**MÉTODOS:**

Estudio transversal descriptivo, del que participaron pediatras (n=132), entrevistados aleatoriamente en evento destinado a la especialidad en agosto de 2012. Se recogieron los datos en una ficha estandarizada y autoadministrada después de la lectura del caso hipotético de un lactante que lloraba excesivamente.

**RESULTADOS:**

Se observó mayor proporción de pediatras del sexo femenino, promedio de edad de 39 años y tiempo de formación profesional mediano de 14 años, siendo que el 52,2% eran portadores del título de especialista por la Sociedad Brasileña de Pediatría. La hipótesis diagnóstica más frecuentemente considerada fue enfermedad del reflujo gastroesofágico (62,9%), seguida por cólicos del lactante (23,5%) y alergia a la proteína de la leche vacuna (6,8%). El examen complementar más frecuentemente indicado fue la pH-metría esofagiana de 24 horas (21,9%). Los medicamentos indicados con mayor frecuencia fueron domperidona en 30,3%, combinación de domperidona con ranitidina (12,1%) y paracetamol (6%).

**CONCLUSIONES:**

En el abordaje del tratamiento del lactante que llora excesivamente, se solicitan frecuentemente exámenes complementarios y se prescriben medicamentos innecesarios.

## Introduction

Newborn crying is a simple behavior, but which involves vast complexity. In the last decades, there were innumerable studies to determine its characteristics, as well as factors associated to its possible etiologies^(^ 1 ^)^.

Excessive crying, given the inherent concern caused in parents, is one of the most frequent reasons of consultation in the first months of life, occurring in 9 to 30% of infants aged lower than 4 months^(^ 1 ^-^ 4 ^)^. The prevalence may vary according to the definition used^(^ 1 ^,^ 4 ^)^.

This phenomenon is usually transitory and is part of the neurologic development, so most infants present episodes of inconsolable crying in the first months life.

According to longitudinal studies, in 5% of infants, crying persists up to 5 months of age^(^ 5 ^)^. The objective of this study was to analyze how pediatricians interpret excessive crying in infants in the first months of life, as well as its respective management, due to the importance of this clinical condition in routine pediatric practice.

## Method

Descriptive cross-sectional study involving a convenience sample consisting of 132 pediatricians randomly included and attendees of a nationwide event on Pediatrics (69^o^ Curso Nestlé), performed in the municipality of Rio de Janeiro in August, 2012. 

The study included pediatric residents, pediatricians with or without a specific pediatric specialty, and general practitioners who were Board Certified by the Brazilian Society of Pediatrics. 

The instrument used for data collection was a standard professional form, which consisted of an initial piece of identification information on sex, age, country of residence, time since graduation in Medical School, degree of specialization in Pediatrics and place of professional practice (clinic, hospital, university and/or public service). The second part consisted of questions regarding the clinical scenario: "two-month-old infant, female, under exclusive breastfeeding, previously healthy, without intercurrences in the neonatal period comes to the pediatrician with maternal complaints of daily excessive crying. Refers the symptoms especially at night with more than 4 hours of progression in the last 3 weeks of life. Presented frequent regurgitations during the day after feedings. The physical examination was appropriate, as well as weight gain and psychomotor development (40g/day)". After reading the case, the following open questions were presented, without alternatives for the answers. Each professional answered freely.

1. Which is the most likely diagnosis in the above case?
2. Would you require an additional exam to better clarify the case? If so, which one?
3. What would be the initial management of this patient?

All 132 forms were returned and fully answered. The answers were interpreted individually, extracting the information, which were included in a spreadsheet. The data, graphs, and tables were generated and analyzed in Microsoft Excel^(r)^ 2007. 

The study was approved by the Research Ethics Committee of Hospital Pequeno Príncipe in the municipality of Curitiba, state of Paraná, and the informed consent was obtained from all participants.

## Results

The general characteristics of the studied population are presented in Table 1. All questionnaires were randomly distributed, which were returned soon after. There was a greater proportion of female pediatricians. Age ranged from 24 to 65 years (mean of 39 years). The time since graduation in Medical School ranged from zero to 37 years (mean of 14 years). Most participants concluded their training in a pediatric residency program and more than half (52.2%) were Board Certified by the Brazilian Society of Pediatrics. Among the interviewees, 112 (85%) did not have a certificate in a specific field of action. Most interviewees (52.2%) lived in the Southeast region of Brazil and 53.7% worked both in the private sector and in the public sector. 

**Table 1:**
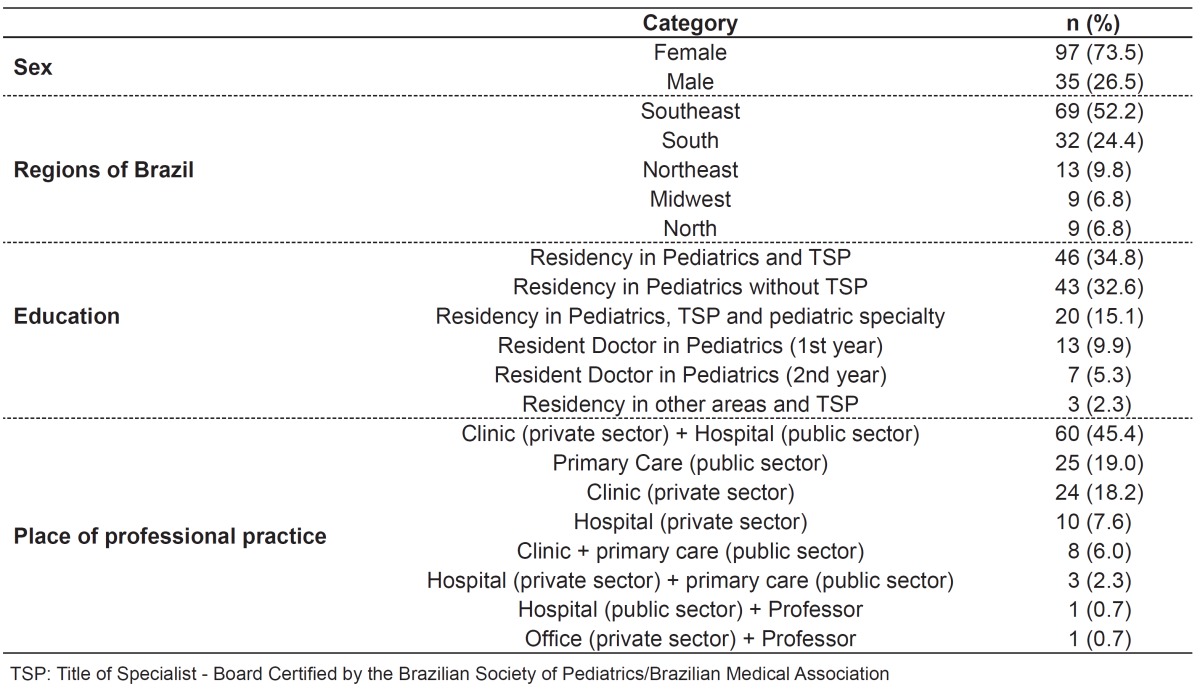
General characteristics of the 132 physicians interviewed

The information on diagnosis, the exam that would be requested, and the management were retrieved from the written answers to the three formulated questions. The three answers were identified on the 132 forms collected.

The diagnostic hypotheses proposed by the respondents are presented in Table 2. Gastroesophageal reflux disease (GERD), followed by infant colic, cow's milk protein allergy (CMPA), and absence of sickness (healthiness) were the most cited diagnosis by physicians. 

**Table 2:**
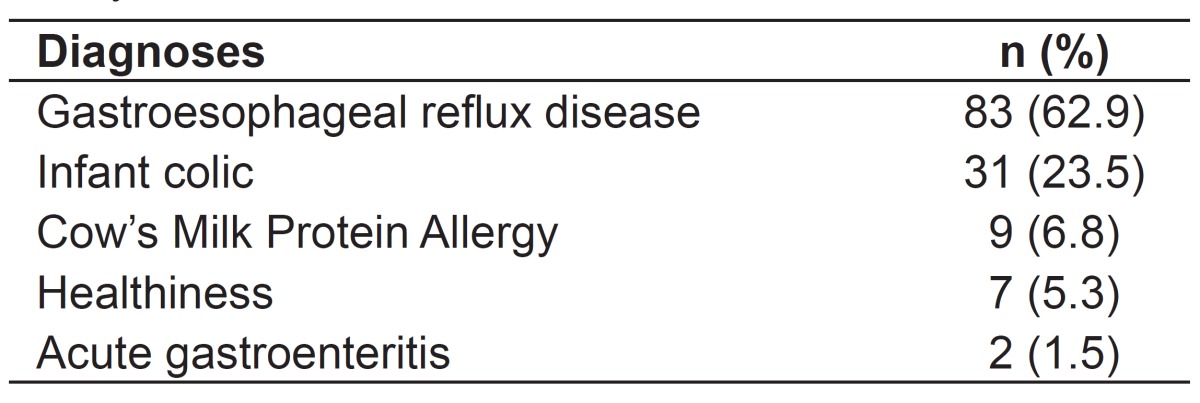
Diagnostic hypotheses established by the 132 physicians surveyed

As to the need for exams to investigate the case, 37.8% of respondents requested an additional exam, and the 24-hour esophageal pH-monitoring was the most cited exam, followed by contrast radiography of the esophagus, stomach, and duodenum (ESD), abdomen ultrasound, upper gastrointestinal endoscopy (UGI), and measurement of Specific IgE against cow's milk (Table 3).

**Table 3:**
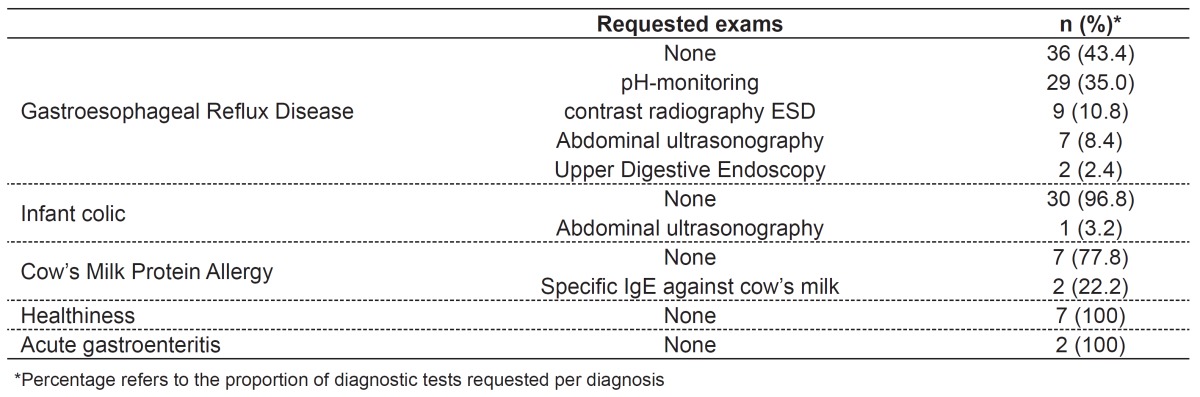
Additional exams requested by the 132 physicians interviewed

The treatment modalities suggested by pediatricians were associated to the diagnosis proposed and are presented in Table 4. Only 20 (15%) physicians did not indicate some therapeutic modality for the management of the reported clinical condition. Among the interviewed physicians, only three (2.2%) cited the instruction of parents about the normalcy of the symptoms as a treatment option. 

**Table 4:**
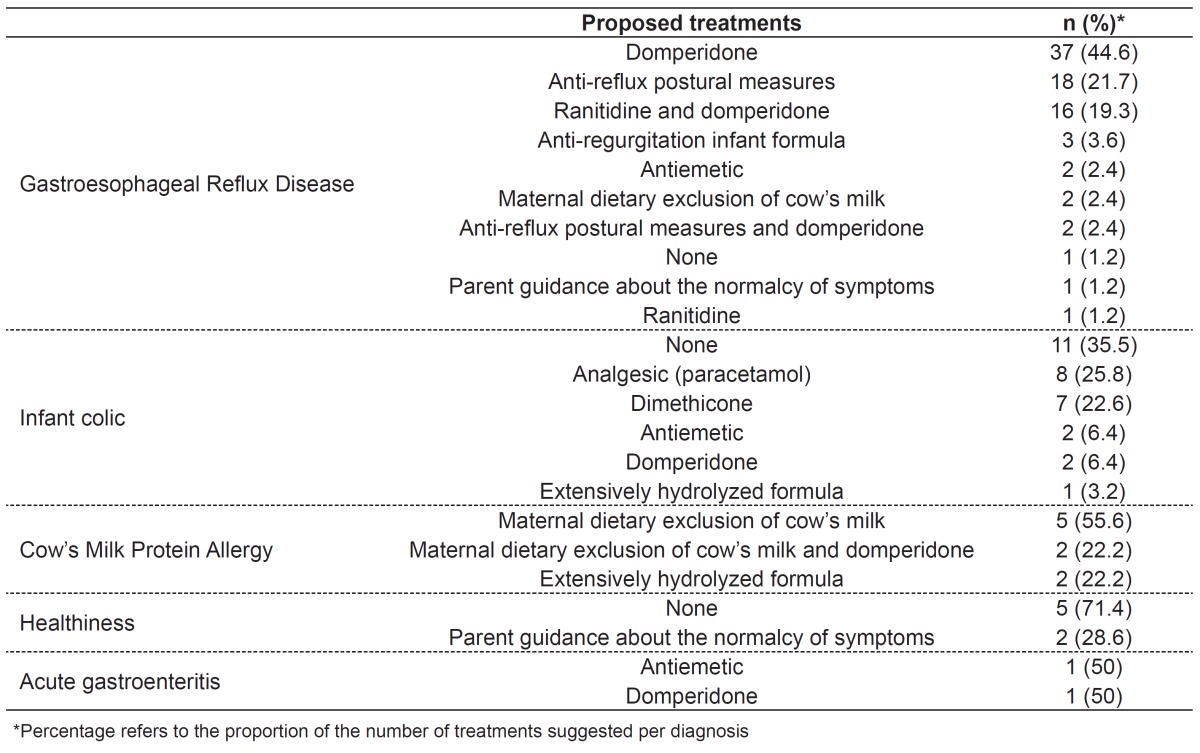
Proposed diagnosis established by the 132 interviewed doctors

When clinical diagnosis was GERD (62.9%), 47 (56.6%) physicians requested additional medical examination (Table3) and 58 (70%) indicated some pharmacological treatment of the case, as shown in Table 4. 

Infant colic was suspected in 31 (23.5%) interviewees and most participants did not request any further exam to complement diagnostic elucidation. However, 19 (61.2%) would indicate some kind of pharmacological treatment, and dimethicone and paracetamol were the medications chosen by these pediatricians to control the symptoms presented by the infant.

## Discussion

Traditionally, excessive crying is defined as a case in which the infant presents irritability, crying and/or agitation for more than 3 hours a day in more than 3 days a week^(^ 6 ^)^. Crying in the first months of life is also contemplated in the Rome III classification as a functional gastrointestinal entity denominated infant colic, practically with the same criteria established by Wessel in the 1950s^(^ 6 ^,^ 7 ^)^. However, there are more subjective definitions when there is maternal observation that the infant is crying or is inconsolable^(^ 4 ^)^_._

Despite the indefinite etiology, some factors have already been implicated, such as: the infant's temperament^(^ 8 ^)^, neurological maturity related to delayed development and maturation of the parasympathetic nervous system, the transition of sleep-wake cycle^(^ 8 ^)^, the poor performance in prenatal care^(^ 1 ^)^ and even cultural organic diseases^(^ 1 ^,^ 2 ^,^ 4 ^,^ 9 ^)^. It is important to mention that only in 5% of cases an underlying organic disease^(^ 1 ^)^ was identified, and, in such cases, normally other factors were associated, such as poor weight gain, changes in the feces and/or developmental delay^(^ 5 ^)^_._

Currently, the most accepted theory is that healthy infants ​​signal the need for a response from their caregiver by changing breathing patterns, color and/or posture variation, manifested by patterns of movement and vocalization of a cry and/or crying, these latter being the highest concerns of caregivers^(^ 5 ^)^. The intensity of the behavior may depend on temperament, neurodevelopmental maturity, ability to adapt to the environment, or unknown factors^(^ 5 ^)^.

Even when considered excessive, crying is a benign entity in most cases, but can lead to short- and long-term consequences, such as early termination of breastfeeding, early introduction of solid foods, frequent change of infant formula, maternal irritability and frustration, reduction of mother-infant interaction, increased risk of physical abuse, behavioral disorders at pre-school age, hyperactivity, and sleep disorders^(^ 8 ^)^. 

In addition to the aforementioned consequences, this clinical condition is often confused with gastrointestinal disorders, such as GERD, and the infant is subjected to unnecessary investigations and potential pharmacological treatments. In this study, GERD was suspected for the majority of respondents (62.9%) and the 24-hour esophageal pH-monitoring was the most requested exam (43%) for diagnostic testing. Despite belonging to the diagnostic arsenal of GERD, this is a valid exam, especially to assess the antisecretory therapy and to investigate atypical manifestations of the disease^(^ 9 ^)^, absent in the clinical case described. Irritability will only be present in a child with GERD if he or she has esophagitis, which is rare in the age range mentioned, and in such cases, upper gastrointestinal endoscopy is the most accurate test to evaluate the esophageal mucosa^(^ 9 ^,^ 10 ^)^.

Although two international consensuses by committees of experts agree that GERD is not a cause of irritability and/or inconsolable crying in the first month of life, many pediatricians attribute a relation between these different situations^(^ 9 ^,^ 10 ^)^. Several studies have demonstrated that the use of acid inhibitors does not lead to the improvement of symptoms in infants with these clinical manifestations^(^ 11 ^,^ 12 ^)^. Furthermore, in recent years, it is noted in medical practice the excessive use of proton pump inhibitors (PPIs) to treat or alleviate intense crying in healthy term children, without signs or symptoms indicating an organic disease. These drugs are not recommended for a child whose only problem is excessive crying, even if it is associated to arching back and refusal to feed^(^ 5 ^,^ 11 ^-^ 15 ^)^. In children with documented GERD, the PPIs have proven effective in reducing acid exposure, but are not able to improve irritability^(^ 12 ^-^ 14 ^)^. Despite the lack of evidence to support its use in the treatment of GERD symptoms in children, PPIs were prescribed to 145 thousand children under 12 months, in 2009, in the United States^(^ 15 ^)^. The use of this medication should be reserved for the treatment of acid-induced lesions, documented by upper endoscopy^(^ 13 ^,^ 15 ^)^. In this study, the interviewed physicians did not mention this medication.

Just as in the crying, the regurgitation and vomiting were common physiological phenomena in children in the first months of life, reaching a maximum of 3 to 4 months of age and, when associated, despite not having a causal relationship, increase the chances of a healthy infant getting at least one medication characterized as anti-reflux^(^ 13 ^,^ 16 ^,^ 17 ^)^. In this study, great part of the interviewed physicians attributed an organic etiology to the excessive crying of an infant with regurgitation, without characteristics of organic disease, adding additional exams and pharmacological treatment for the management of the case. It should be noted that a significant number of pediatricians prescribed domperidone and ranitidine, including combined, for the management of the chief complaint, crying.

The key-question for the pediatrician is to distinguish the clinical manifestations of physiological gastroesophageal reflux (GER) from GERD, to identify the patients who need investigation and/or treatment^(^ 18 ^)^. The clinical history and physical examination, with attention to warning signs, are usually sufficient to allow the clinician to establish the difference^(^ 19 ^)^. Parental guidance and clarification are essential^(^ 19 ^)^. The spontaneous resolution of GER is common and the evolution is generally benign, with low incidence of complications^(^ 18 ^,^ 20 ^)^. Around 70-85% of children have regurgitations in the fist 2 months of life and it resolves without intervention in 95% of children until 1 year of age^(^ 20 ^)^. Therefore, the prolonged or repeated use of pharmacological therapy should not be prescribed before diagnostic confirmation, especially in infants^(^ 18 ^)^.

It is crucial that pediatricians learn to recognize situations that are considered physiological to minimize unnecessary additional investigations as well as to decrease the anxiety of parents, explaining the benignity of the condition.

The results of this study allow us to conclude that the respondents demonstrated inadequacy in addressing the child that cries excessively in the first months of life, as well as in the investigation and management of gastrointestinal conditions in childhood, such as GERD and CMPA.

Excessive crying in infants demand attention by pediatricians and longer outpatient visits. However, non-pharmacological guidelines given by an experienced professional regarding breastfeeding, as well as information about the absence of organic disease, have good results^(^ 16 ^,^ 17 ^)^. The doctor should also pay attention to maternal mental health and the repercussions of this situation on the family context^(^ 17 ^)^.

Considering that the sample evaluated in this investigation was gathered in a pediatric update event, and understanding that professionals that seek these activities are usually more interested in continuing education programs, the results cannot be generalized to the whole population of pediatricians. It is possible that samples including physicians who do not attend continuing education activities may reveal a number of unsubstantiated practices for infant crying, which may be a step within normal development. 

These data emphasize the need for the development of educational strategies to enhance the knowledge of these professionals, in order to avoid excessive additional investigations and the prescription of medications with potential adverse effects and no benefits in the natural evolution of the crying infant.
