# Supplementary material for: Pediatrician's knowledge on the management of the infant who cries excessively in the first months of life
Source: Rev Paul Pediatr. 2014 Jun;32(2):187–92. doi: 10.1590/0103-0582201432218713 (PMC4183018; doi:10.1590/0103-0582201432218713)
Supplement: Translation

# Translation: Conhecimentos do pediatra sobre o manejo do lactente que chora excessivamente nos primeiros meses de vida

**Authors:** Ana Carolina C. Marcon, Mário César Vieira, Mauro Batista de Morais

**Affiliations:** 1 Hospital Pequeno Príncipe, Curitiba, PR, Brasil; 2 Escola Paulista de Medicina da Universidade Federal de São Paulo (Unifesp), São Paulo, SP, Brasil

**Keywords:** choro, lactente, educação em saúde

## Abstract

**OBJETIVO:**

Avaliar a atitude, a prática e o conhecimento de pediatras sobre o manejo do lactente que chora excessivamente nos primeiros meses de vida.

**MÉTODOS:**

Estudo transversal descritivo, do qual participaram pediatras (n=132), entrevistados aleatoriamente em evento destinado a especialidade em agosto de 2012. Coletaram-se os dados em uma ficha padronizada e autoadministrada após a leitura do caso hipotético de um lactente que chorava excessivamente.

**RESULTADOS:**

Observou-se maior proporção de pediatras do sexo feminino, média de idade de 39 anos e tempo de formação profissional médio de 14 anos, sendo que 52,2% eram portadores do título de especialista pela Sociedade Brasileira de Pediatra. A hipótese diagnóstica mais frequentemente considerada foi doença do refluxo gastroesofágico (62,9%), seguida por cólica do lactente (23,5%) e alergia à proteína do leite de vaca (6,8%). O exame complementar mais frequentemente indicado foi a pH-metria esofagiana de 24 horas (21,9%). As medicações indicadas com maior frequência foram domperidona em 30,3%, combinação de domperidona com ranitidina (12,1%) e paracetamol (6%).

**CONCLUSÕES:**

Na abordagem do lactente que chora excessivamente, solicitam-se frequentemente exames complementares e prescrevem-se medicamentos desnecessários.

## Introdução

O choro do lactente é um comportamento simples, mas que se reveste de enorme complexidade. Nas últimas décadas, foram inúmeros os estudos para se determinarem as suas características, bem como os fatores associados e suas possíveis etiologias^(^ ^1^ ^)^.

O choro excessivo, dada a inerente preocupação provocada nos pais, constitui um dos motivos mais frequentes de consulta nos primeiros meses de vida, ocorrendo em 9-30% dos lactentes com idade inferior a quatro meses^(^ ^1^ ^-^ ^4^ ^)^. A prevalência pode variar conforme a definição utilizada^(^ ^1^ ^,^ ^4^ ^)^.

Esse fenômeno costuma ser transitório e faz parte do desenvolvimento neurológico, sendo que a maioria dos lactentes apresenta episódios de choro inconsolável nos primeiros meses de vida.

Segundo estudos longitudinais, em 5% dos lactentes, o choro persiste até os cinco meses de idade^(^ ^5^ ^)^. O objetivo deste trabalho foi analisar de que forma os médicos pediatras interpretam o choro excessivo do lactente nos primeiros meses de vida, bem como o seu respectivo manejo, devido à importância dessa situação clínica na prática pediátrica de rotina.

## Método

Estudo transversal descritivo envolvendo uma amostra de conveniência constituída por 132 pediatras incluídos aleatoriamente e participantes de um evento de Pediatria de âmbito nacional, realizado na cidade do Rio de Janeiro em agosto de 2012. 

Incluíram-se médicos residentes em Pediatria, pediatras com ou sem área de atuação (especialidades pediátricas) e médicos generalistas com título de especialista em Pediatria conferido pela Sociedade Brasileira de Pediatria (SBP). 

O instrumento utilizado para a coleta de dados dos profissionais foi uma ficha padronizada, que constava de uma parte inicial de identificação com informações sobre sexo, idade, região do país de residência, tempo de graduação no curso de Medicina, grau de especialização em Pediatria e local de atuação profissional (consultório, hospital, universidade e/ou serviço público). A segunda parte foi constituída por perguntas a respeito da seguinte situação clínica: "lactente de dois meses de idade, sexo feminino, em aleitamento materno exclusivo, previamente hígida e sem intercorrências no período neonatal vem ao pediatra com queixa materna de choro excessivo diariamente. Refere o sintoma principalmente no período noturno com mais de quatro horas de evolução nas últimas três semanas de vida. Apresentava regurgitações frequentes durante o dia após as mamadas. O exame físico era adequado, bem como o desenvolvimento neuropsicomotor e o ganho ponderal (40g/dia)". Após a leitura do caso, apresentaram-se as seguintes perguntas abertas, não tendo sido oferecidas alternativas para as respostas. Cada profissional respondeu utilizando redação livre.

1. Qual o diagnóstico mais provável do caso descrito?
2. Você solicitaria algum exame complementar para melhor esclarecimento do caso? Se sim, qual?
3. Qual seria o manejo inicial desse paciente?

Todas as 132 fichas distribuídas foram devolvidas e respondidas integralmente. Interpretaram-se as respostas individualmente, extraindo-se as informações, que foram incluídas em uma planilha. Os dados, gráficos e tabelas foram gerados e analisados no programa Microsoft Excel^(r)^ 2007. 

O estudo foi aprovado pelo Comitê de Ética em Pesquisa do Hospital Pequeno Príncipe em Curitiba, PR, obtendo-se o consentimento esclarecido por escrito de todos os participantes.

## Resultados

As características gerais da população estudada estão apresentadas na Tabela 1. Distribuíram-se todos os questionários aleatoriamente, os quais foram entregues em seguida. Observa-se maior proporção de pediatras do sexo feminino. A idade variou de 24 a 65 anos (média de 39 anos). O tempo de graduação em Medicina variou de zero e 37 anos (média de 14 anos). A maioria dos participantes concluiu treinamento em programa de residência médica em Pediatria e mais da metade (52,2%) era portador do título de especialista pela SBP. Dos entrevistados, 112 (85%) não possuíam certificado em área de atuação. A maioria dos entrevistados (52,2%) residia na região Sudeste do Brasil e 53,7% trabalhavam tanto no setor privado quanto no setor público de saúde. 

**Tabela 1:** Características gerais dos 132 médicos entrevistados

As informações sobre o diagnóstico, o exame que solicitaria e as condutas foram retiradas das respostas discursivas às três perguntas formuladas. Identificaram-se as três respostas nas 132 fichas coletadas.

As hipóteses diagnósticas propostas pelos entrevistados são apresentadas na Tabela 2. A doença do refluxo gastroesofágico (DRGE), seguida por cólica do lactente, alergia à proteína do leite de vaca (APLV) e ausência de doença (higidez) foram os diagnósticos mais citados pelos médicos entrevistados. 

**Tabela 2:** Hipóteses diagnósticas estabelecidas pelos 132 médicos entrevistados

Quanto à necessidade de exames para investigar o caso, 37,8% dos entrevistados solicitaram algum exame complementar, sendo a pH-metria esofagiana de 24 horas o mais citado, seguido por radiografia contrastada de esôfago-estômago e duodeno (EED), ultrassonografia de abdome, endoscopia digestiva alta (EDA) e dosagem de IgE específico para leite de vaca (Tabela 3).

**Tabela 3:** Exames complementares por diagnóstico solicitados pelos 132 médicos entrevistados

Os tratamentos sugeridos pelos pediatras associaram-se aos diagnósticos propostos e são apresentados na Tabela 4. Apenas 20 (15%) médicos não indicaram alguma modalidade terapêutica para o manejo do quadro clínico relatado. Entre os médicos entrevistados, apenas três (2,2%) citaram como opção de tratamento a orientação aos pais quanto à normalidade dos sintomas apresentados. 

**Tabela 4:** Tratamento proposto por diagnóstico estabelecido pelos 132 médicos entrevistados

Quando a suspeita clínica foi DRGE (62,9%), 47 (56,6%) médicos solicitaram exame complementar (Tabela 3) e 58 (70,0%) indicaram algum tratamento farmacológico para o caso, conforme apresentado na Tabela 4. 

A cólica do lactente foi a suspeita diagnóstica de 31 (23,5%) entrevistados e a maioria não solicitou nenhum exame complementar para elucidação diagnóstica. No entanto, 19 (61,2%) indicariam algum tratamento farmacológico, sendo a dimeticona e o paracetamol as medicações escolhidas por esses pediatras para controlar os sintomas apresentados pelo lactente.

## Discussão

Tradicionalmente, define-se como choro excessivo o caso em que o lactente apresenta irritabilidade, choro e/ou agitação com duração superior a três horas diárias, em mais de três dias por semana^(^ ^6^ ^)^. O choro nos primeiros meses de vida também é contemplado na classificação Roma III como uma entidade gastrintestinal funcional com a denominação de cólica do lactente, praticamente com os mesmos critérios estabelecidos por Wessel na década de 1950^(^ ^6^ ^,^ ^7^ ^)^. No entanto, existem definições mais subjetivas, quando há constatação materna de que o lactente chora muito ou é inconsolável^(^ ^4^ ^)^_._

Apesar de etiologia indefinida, já se implicaram fatores como: o temperamento do lactente^(^ ^8^ ^)^, a maturidade neurológica relacionada ao atraso de desenvolvimento e de maturação do sistema nervoso parassimpático, a transição do ciclo sono-vigília^(^ ^8^ ^)^, a deficiente prestação de cuidados parentais^(^ ^1^ ^)^ e até mesmo as diferenças culturais^(^ ^1^ ^,^ ^2^ ^,^ ^4^ ^,^ ^9^ ^)^. É importante ressaltar que apenas em 5% dos casos pode-se identificar doença orgânica subjacente^(^ ^1^ ^)^, sendo que, nesses casos, normalmente outros fatores estão associados, como baixo ganho ponderal, alterações nas fezes e/ou atraso do desenvolvimento neuropsicomotor^(^ ^5^ ^)^.

Atualmente, a teoria mais aceita é a de que lactentes saudáveis ​​sinalizam a necessidade de uma resposta de seu cuidador com mudança do padrão respiratório, variação de cor e/ou postura, manifestando-se por padrões de movimento e vocalizações de um grito e/ou choro, sendo estes últimos os que causam maior preocupação dos seus cuidadores^(^ ^5^ ^)^. A intensidade do comportamento pode depender do temperamento, da maturidade do desenvolvimento neurológico, da capacidade de se adaptar ao meio ambiente ou de fatores desconhecidos^(^ ^5^ ^)^.

Mesmo quando considerado excessivo, o choro é uma entidade benigna na maioria dos casos, mas pode conduzir a consequências de curto e longo prazo, como interrupção prematura do aleitamento materno, introdução precoce de alimentos sólidos, mudança frequente da fórmula infantil, irritabilidade e frustração materna, diminuição da interação mãe-lactente, aumento do risco de abuso físico, alterações comportamentais na idade pré-escolar, hiperatividade e distúrbios do sono^(^ ^8^ ^)^. 

Além das consequências citadas, frequentemente se confunde essa situação clínica com afecções gastrintestinais, como a DRGE, e o lactente é submetido a investigações complementares e a tratamentos farmacológicos desnecessários e com riscos potenciais. Neste estudo, a DRGE foi a suspeita diagnóstica para a maioria dos entrevistados (62,9%) e a pH-metria esofagiana de 24 horas foi o exame mais solicitado (43%) para elucidação diagnóstica. Apesar de pertencer ao arsenal diagnóstico da DRGE, esse é um exame válido principalmente para se avaliar a terapia antissecretora e investigar manifestações atípicas da doença^(^ ^9^ ^)^, ausentes no caso clínico descrito. A irritabilidade só estará presente em uma criança com DRGE caso ela tenha esofagite, o que é raro na faixa etária citada e, nesses casos, a endoscopia digestiva alta é o exame de melhor acurácia para avaliar a mucosa esofagiana^(^ ^9^ ^,^ ^10^ ^)^.

Embora dois consensos internacionais por comitês de especialistas concordem que a DRGE não é uma causa de irritabilidade e/ou choro inconsolável nos primeiros meses de vida, muitos pediatras atribuem relação entre essas situações distintas^(^ ^9^ ^,^ ^10^ ^)^. Diversos estudos têm demonstrado que o uso de inibidores de ácido não induz à melhora de sintomas em lactentes com essas manifestações clínicas^(^ ^11^ ^,^ ^12^ ^)^. Além disso, nos últimos anos, nota-se na prática médica o uso excessivo de inibidores de bomba de próton (IBP) para tratar ou amenizar o choro intenso de crianças a termo, saudáveis e sem outros sinais ou sintomas que apontem para uma doença orgânica. Esses fármacos não são indicados para uma criança cujo único problema é o choro excessivo, mesmo que este se associe ao arqueamento para trás e à recusa alimentar^(^ ^5^ ^,^ ^11^ ^-^ ^15^ ^)^. Nas crianças com DRGE documentada, os IBP mostram-se eficazes em reduzir a exposição ácida, mas não são capazes de melhorar a irritabilidade^(^ ^12^ ^-^ ^14^ ^)^. Apesar da falta de evidências para apoiar seu uso no tratamento dos sintomas da DRGE em crianças, os IBP foram prescritos para 145 mil crianças com menos de 12 meses, em 2009, nos Estados Unidos^(^ ^15^ ^)^. O uso dessa medicação deve ser reservado para o tratamento de lesões induzidas por ácido, documentadas por endoscopia digestiva alta^(^ ^13^ ^,^ ^15^ ^)^. Neste estudo, essa medicação não foi citada pelos médicos entrevistados.

Assim como o choro, as regurgitações e os vômitos frequentes são fenômenos fisiológicos em crianças nos primeiros meses de vida, atingindo um máximo de três a quatro meses de idade e, quando associados, apesar de não terem relação causal, aumentam a chance de um lactente não doente acabar recebendo pelo menos uma medicação caracterizada como antirrefluxo^(^ ^13^ ^,^ ^16^ ^,^ ^17^ ^)^. Neste estudo, grande parte dos médicos entrevistados atribuiu etiologia orgânica ao choro excessivo de um lactente com regurgitações, sem características de doença orgânica, agregando exames complementares e tratamento farmacológico para o manejo do caso. Deve-se destacar que um número expressivo de pediatras prescreveu a domperidona e a ranitidina, inclusive combinadas, para o manejo da principal queixa, o choro.

A questão-chave para o pediatra é distinguir as manifestações clínicas do refluxo gastroesofágico fisiológico (RGE) da DRGE, para se identificarem os pacientes que necessitam de investigação e/ou tratamento^(^ ^18^ ^)^. A história clínica e o exame físico, com atenção aos sinais de alerta, geralmente são suficientes para permitir que o clínico estabeleça a diferença^(^ ^19^ ^)^. A orientação e o esclarecimento aos pais são essenciais^(^ ^19^ ^)^. A resolução espontânea do RGE é comum e a evolução é geralmente benigna, com baixa incidência de complicações^(^ ^18^ ^,^ ^20^ ^)^. Cerca de 70-85% das crianças têm regurgitações nos primeiros dois meses de vida e isso se resolve sem intervenção em 95% das crianças até um ano de idade^(^ ^20^ ^)^. Portanto, o uso prolongado ou repetido de tratamento farmacológico não deve ser prescrito antes da confirmação diagnóstica, especialmente em lactentes^(18) ^.

É fundamental que o pediatra saiba reconhecer situações consideradas fisiológicas da criança para minimizar investigações complementares desnecessárias, bem como diminuir a ansiedade dos pais, explicando a benignidade do quadro.

Os resultados deste trabalho permitem concluir que os profissionais entrevistados demonstraram inadequação na abordagem à criança que chora excessivamente nos primeiros meses de vida, bem como na investigação e no manejo de situações gastrintestinais na infância, como a DRGE e a APLV.

O choro excessivo do lactente demanda atenção do pediatra e maior tempo de consulta ambulatorial. Todavia, orientações não farmacológicas dadas por um profissional experiente quanto ao aleitamento materno, bem como informações sobre a ausência de doença orgânica, têm bons resultados^(^ ^16^ ^,^ ^17^ ^)^. O médico deve se atentar também à saúde mental materna e às repercussões dessa situação no contexto familiar^(^ ^17^ ^)^.

Considerando-se que a amostragem avaliada neste inquérito constituiu-se em um evento de atualização pediátrica, entendendo-se que profissionais que busquem essas atividades normalmente são mais interessados em programas de educação continuada, os resultados não podem ser generalizados para a população total de pediatras. É possível que amostras incluindo médicos que não frequentam atividades de educação continuada revelem um número de condutas não fundamentadas para lactentes com choro, o qual pode constituir uma etapa dentro do desenvolvimento normal. 

Esses dados ressaltam a necessidade de se elaborarem estratégias educacionais que ampliem os conhecimentos desses profissionais, para evitar investigações complementares excessivas e a prescrição de medicamentos com potenciais eventos adversos e sem benefícios na evolução natural do choro do lactente.
